# Awareness and use of short-fill e-liquids by youth in England in 2021: findings from the ITC Youth Tobacco and Vaping Survey

**DOI:** 10.1136/tc-2022-057871

**Published:** 2023-05-02

**Authors:** Eve Taylor, Katherine East, Jessica L Reid, David Hammond

**Affiliations:** 1 Addictions Department, King's College London, London, UK; 2 School of Public Health Sciences, University of Waterloo, Waterloo, Ontario, Canada; 3 School of Public Health and Health Systems, University of Waterloo, Waterloo, Ontario, Canada

**Keywords:** nicotine, electronic nicotine delivery devices, public policy

## Abstract

**Background:**

Refillable e-cigarettes were popular among youth in England in 2021. The UK Tobacco and Related Products Regulations (TRPR) limits e-liquids to 20 mg/mL of nicotine in a 10 mL bottle. Short-fill e-liquids, which are not covered by TRPR regulations, are typically nicotine-free and come in larger, underfilled bottles allowing customisation with the addition of ‘nicotine shots’. This paper investigates awareness, use, and reasons for use of short-fill e-liquids among youth in England.

**Methods:**

Data are from the online 2021 International Tobacco Control Youth Survey, comprising 4224 youth (aged 16–19 years) in England. Weighted logistic regression models investigated associations between awareness and past 30-day use of short-fills by smoking status, vaping status, nicotine strength vaped and participant demographics. Reasons for use were also reported.

**Results:**

Approximately one-quarter (23.0%) of youth in England reported awareness of short-fill e-liquids. Among youth who had vaped in the past 30 days, 22.1% had used short-fills in the past 30 days; use was most prevalent among those who were also smoking (43.2%) and those who reported usually vaping nicotine concentrations of 2.1% (21 mg/mL) or more (40.8%). ‘Convenience of a bigger bottle’ was the most selected reason for use (45.0%), followed by ‘less expensive than regular e-liquids’ (37.6%).

**Conclusions:**

Awareness of short-fills was common among youth in 2021, including among those who had never vaped or smoked. Among youth who vaped in the past 30 days, short-fill use was more prevalent among those who also smoked and those who vaped nicotine-containing e-liquids. Integration of short-fill products into existing e-cigarette regulations should be considered.

**WHAT IS ALREADY KNOWN ON THIS SUBJECT**

- ‘Short-fills’ are large, typically underfilled, bottles of nicotine-free e-liquids that can be mixed with nicotine shots to customise an e-liquid.

***W*HAT THIS STUDY ADDS**

- Short-fills sit outside the UK Tobacco and Related Products Regulations, and little is known about their use among youth.

**HOW THIS STUDY MIGHT AFFECT RESEARCH, POLICY OR PRACTICE**

- We find that, short-fill use is common among youth who vape in England.
- Future research should examine how youth are accessing short-fill e-liquids and consider the need to integrate nicotine-free and nicotine-containing vaping product policy.

## Introduction

 E-cigarette use (vaping) has increased among youth in England with 15.8% of those aged 11–17 years trying vaping in 2022, compared with 13.9% in 2020.^1^ Although e-cigarettes and e-liquids can only legally be sold to people aged 18 years or over in England, 46.5% of those aged 11–17 years report purchasing products from shops.^1^

In 2021, refillable tank e-cigarettes were the most used vaping device among youth in England.^2^ ‘Short-fill’ e-liquids are nicotine-free liquids that come in larger, underfilled bottles (typically 50 mL), which consumers can use nicotine-free, or mix with ‘nicotine shots’. Short-fills offer consumers larger e-liquid quantities and the ability to mix custom nicotine concentrations, flavours, and propylene glycol/vegetable glycerine (PG/VG) ratios, often for a lower price-per-mL than premixed liquids. As short-fill e-liquids do not contain nicotine, they are not regulated by the UK Tobacco and Related Products Regulations (TRPR) and so do not include nicotine warning labels and are not limited to 10 mL bottles.^3^ However, ‘nicotine shots’ are regulated by the TRPR, and therefore, like other nicotine-containing e-liquids, packaging should contain nicotine warning labels and require labelling of nicotine content, ingredients and relevant hazard symbols. Nicotine-containing e-liquids in the UK can be a maximum 10 mL in bottle size and have a maximum nicotine strength of 20 mL/mg.^3 4^ However, it is not uncommon for illicit products to be easily available, for example, disposable vapes with nicotine >20 mg/mL have been documented for sale.^5^

Little is understood about if, or why, youth use short-fill e-liquids. Therefore, this paper investigates awareness, prevalence of use and reasons for use of short-fill e-liquids among youth in England.

## Methods

*Participants:* participants were from wave 5 (August 2021) of the International Tobacco Control Youth Tobacco and Vaping Survey, a repeated cross-sectional online survey of youth aged 16–19 years in England, Canada and the USA. A full description of the study methods can be found in the Technical Reports.^6^ Briefly, respondents were recruited through the Nielsen Consumer Insights Global Panel directly or through their parents and completed an online survey.

A total of 4500 surveys were completed in England. Those who provided incomplete or invalid data (n=89), failed data quality checks (n=95), were missing data on smoking status (n=14), refused to answer or reported to not know their race/ethnicity (n=63) or refused to answer questions on short-fills (n=4) or nicotine strength (n=11) were removed, for a final analytical sample of n=4224.

### Measures

All youth were asked about their awareness of short-fills. Responses were coded ‘aware of short-fills’, and ‘other’ (not aware, do not know).

Youth who currently vaped and reported awareness of short-fills were asked if they had used short-fills in the past 30 days. Responses were coded ‘yes’ and ‘other’ (no, do not know). In deviation from preregistered analysis, youth who had vaped in the past 30 days who were not aware of short-fills were also coded as ‘other’.^7^

Participants who had used a short-fill in the past 30 days were asked their reason(s) for use (select all that apply): ‘to use nicotine-free e-liquid’, ‘to get a higher nicotine concentration (>20 mg/mL)’, ‘less expensive than regular e-liquid’, ‘convenience of a bigger bottle’, ‘to get a custom flavour or PG/VG mix’, ‘other’, ‘do not know’.

Vaping and smoking status, and dual use, were assessed. Nicotine concentration vaped was coded to reflect use of: nicotine-free (0 mg/mL), nicotine levels lower than the legal maximum (1–19 mg/mL), maximum nicotine levels legally available (20 mg/mL), nicotine levels above the maximum limit (≥21 mg/mL). Race/Ethnicity, age and sex were also assessed (see online supplemental table 1).

## Analysis

Analyses were pre-registered.^7^

Cross-sectional survey weights, based on population estimates for sociodemographic variables and calibrated to wave 1 proportions for key variables, were used in all analyses.^6^

Weighted proportions and percentages were calculated for outcome and demographic variables.

The first (1A, 1B), weighted logistic regressions models included smoking status, vaping status, age, sex and race/ethnicity as cross-sectional factors associated with (a) awareness of short fills among all participants and (b) use of short-fills among youth who had vaped in the past 30 days.

Sensitivity analyses were conducted for model 1A, to assess how associations differed when smoking and vaping is combined into dual use.

The second models (2A, 2B) were conducted only among youth who had vaped in the past 30 days. Weighted logistic regressions models included current e-liquid nicotine concentration, smoking status and age, sex and race/ethnicity as cross-sectional factors associated with (a) awareness of short fills and (b) use of short-fills.

Reasons for use were not modelled as an outcome due to low cell counts.

## Results

Table 1 presents sample characteristics, awareness and use of short-fills.

**Table 1:** Awareness and use of short-fills by respondent characteristics (ITC Youth Survey, England, 2021, weighted % (n))

Outcome		Aware of short-fills			Used a short-fill in the past 30 days		
Sample	Totaln=4224	Among all respondents(n=4224)			Among youth who vaped in the past 30 days(n=747)		
	% (n)	% (n)	AOR (95% CI)	P value	% (n)	AOR (95% CI)	P value
**Model 1**							
**Total**		23.0 (974)			22.1 (150)		
***Vaping***							
Never vaped	57.5 (2429)	18.4 (471)	1	Ref	–	–	–
Ever vaped	24.8 (1048)	21.8 (219)	0.93 (0.73 to 1.18)	0.550	–	–	–
Vaped in the past 30 days	17.7 (747)	41.9 (284)	2.14 (1.67 to 2.74)	**<0.001**	22.1 (150)	–	–
***Smoking***							
Never smoked	58.9 (2486)	18.1 (471)	1	Ref	5.3 (6)	1	
Ever smoked	33.9 (1433)	27.0 (370)	1.30 (1.05 to 1.61)	**0.015**	19.5 (83)	4.58 (1.60 to 13.14)	**0.005**
Currently smoking	7.2 (305)	50.9 (133)	3.13 (2.26 to 4.33)	**<0.001**	43.2 (61)	13.7 (4.56 to 40.93)	**<0.001**
**Model 2**		Among youth who vaped in the past 30 days (n*=*747)			Among youth who vaped in the past 30 days (n*=*747)		
***Nicotine concentration***							
0% (0 mg/mL)	40.7 (304)	31.8 (88)	**1**	**Ref**	11.9 (33)	1	Ref
0.1%–1.9% (1–19 mg/mL)	27.6 (206)	54.1 (98)	1.68 (1.06 to 2.69)	**0.029**	32.5 (59)	2.40 (1.32 to 4.39)	**0.004**
2.0% (20 mg/mL)	8.0 (60)	45.8 (23)	1.41 (0.74 to 2.69)	0.294	31.7 (15)	2.64 (1.14 to 6.13)	**0.024**
≥2.1% (≥21 mg/mL)	11.4 (85)	61.7 (55)	2.22 (1.23 to 3.99)	**0.008**	40.8 (37)	3.16 (1.53 to 6.49)	**0.002**
Do not know	12.3 (92)	24.3 (24)	0.59 (0.31 to 1.10)	0.097	7.4 (6)	3.16 (1.53 to 6.49)	0.119

All analyses were weighted.

Model 1 was adjusted for age, sex, race/ethnicity, smoking, vaping.

Model 2 included only youth who had vaped in the past 30 days and was adjusted for age, sex, race/ethnicity, smoking and nicotine concentration.

AORadjusted ORITCInternational Tobacco ControlRefreference

### Awareness of short-fills

Almost one-quarter (23.0%) of youth were aware of short-fill e-liquids. More youth who had vaped in the past 30 days were aware of short-fills than youth who had never vaped (41.9% vs 18.4%). Youth who currently smoked (50.9%) or had ever smoked (27.0%) also had greater awareness than those who had never smoked (18.1%) (table 1). Awareness was greater among youth aged 18–19 years than those aged 16–17 years, and there was little difference by sex or race/ethnicity (online supplemental table 2).

When sensitivity analyses were conducted to explore dual use of smoking and vaping, awareness was significantly greater among youth who vaped and smoked (64.7%), than those who exclusively vaped (35.9%), exclusively smoked (34.7%), had formerly vaped/smoked (20.4%) or had never vaped/smoked (17.9%) (online supplemental table 3).

Among youth who had vaped in the past 30 days, awareness was greater among those who currently used e-liquids that were 1–19 mg/mL (54.1%) or ≥21 mg/mL (61.7%) nicotine compared with those who reported currently using nicotine-free e-liquids (31.8%). There was little significant difference between reporting using nicotine-free e-liquids (31.8%) and using 20 mg/mL of nicotine (45.8%) in adjusted models, potentially due to low sample sizes of youth using 20 mg/mL (adjusted OR 1.41, 95% CI 0.74 to 2.69) (table 1).

#### Past 30-day use of short-fills

Among youth who had vaped in the past 30 days, 22.1% had used a short-fill in the past 30 days. More youth who had vaped in the past 30 days and currently smoked (43.2%) had used short-fills than those who had never smoked (5.3%) (table 1). More males (30.2%) had used a short-fill, compared with females (14.5%), but there were no significant differences by age or race/ethnicity (online supplemental table 2).

Among youth who had vaped in the past 30 days, more participants who vaped nicotine strengths of 1–19 mg/mL (32.5%), 20 mg/mL (31.7%) or ≥21 mg/mL (40.8%) used short-fills, compared with those who used nicotine-free e-liquids (11.9%) (table 1).

##### Reasons for use

Among youth who had used a short-fill in the past 30 days, convenience of a bigger bottle (45.0%) was the most commonly selected reason for use, followed by short-fills being less expensive (37.6%), to get customisation of flavour or PG/VG (34.5%), to get a higher nicotine concentration (>20 mg/mL) (27.6%) and ability to use short-fills as a nicotine-free e-liquid (25.4%) (online supplemental table 4).

## Discussion

Just under one-quarter of youth (23%) surveyed reported awareness of short-fill e-liquids while just under one-quarter (22%) who had vaped in the past 30 days had used short-fill e-liquids.

Findings suggest that the prevalence of short-fill use is not uncommon among youth in England, potentially indicating the need for short-fills, and other nicotine-free products, to be integrated into existing monitoring and regulation such as the TRPR. Ensuring that policy introduced to reduce overall youth use would also apply to these products. Any regulatory changes should avoid unintended consequences among adults who may also use short-fills to quit or reduce their smoking, especially as these may be more economical products for disadvantaged smokers. Moreover, in combination with findings concerning the rapid growth in disposable use,^1^ ice flavours,^8^ and flavoured nicotine pouches;^9^ our findings also highlight an overall need for continual monitoring of products used by youth in an ever changing market.

As ‘nicotine shots’ are regulated by the TRPR, nicotine concentrations cannot legally exceed 20 mg/mL in the UK^4^; therefore, concentrations >20 mg/mL should not be legally obtainable. However, ‘to get a higher nicotine concentration (>20 mg/mL)’ was selected by just under one-third of youth who used short-fills. Disposable vapes >20 mg/mL have been found for sale in the UK; therefore, it is possible that ‘nicotine shots’ >20 mg/mL are also obtainable in the UK.^5^ However, youth are often unaware of the nicotine content of e-liquids,^10^ and have difficulty understanding nicotine concentrations of e-liquid.^11^ Also, there may also be confusion among youth with how nicotine dilution works, or that short-fills are nicotine-free; indeed, adverts for short-fills are not always clear about how nicotine is added to the products.^12^

In summary, among youth in England, awareness of short-fill e-liquids, as well as use among those who vape, is common. Findings highlight the importance of investigating the types of vaping products that youth use when considering prevalence of vaping. Future research should examine how youth are accessing products, and any potential misunderstandings concerning nicotine content and strength.

## supplementary material

10.1136/tc-2022-057871 online supplemental file 1
