# Supplementary material for: Awareness and use of short-fill e-liquids by youth in England in 2021: findings from the ITC Youth Tobacco and Vaping Survey
Source: Tob Control. 2023 May 2;33(5):e057871. doi: 10.1136/tc-2022-057871 (PMC10620100; doi:10.1136/tc-2022-057871)
Supplement: online supplemental file 1 [file tc-33-5-s001.pdf]

**Supplementary Table 1: Measures assessing short-fills, vaping and smoking status.**
Awareness of short-fills:
All respondents were asked, “Have you ever heard of a “shortfill” e-liquid (i.e., a nicotine-free e-liquid that you mix with a separate bottle of nicotine)?”<br>Responses were coded:<br>• Yes<br>• Other (No, don’t know)<br>Refusals were excluded
Past 30-day short-fill use:
Youth who vaped in the past 30-days who were aware of short-fills were asked, “In the past 30 days, have you <u>used</u> a “shortfill” e-liquid (i.e., a nicotine-free e-liquid that you mix with a separate bottle of nicotine)?”<br>Responses were coded:<br>• Yes<br>• Other (No, don’t know)*<br>Refusals were excluded<br>*In deviation from pre-registered analysis, youth who vaped in the past 30-days who were not aware of short-fills were also coded as ‘Other’.
Reason(s) for short-fill use:
Those who had used a short-fill in the past 30 days were asked, “Have you used “shortfill” e-liquids for any of the following reasons? (select all that apply)”<br>Options available:<br>• To use nicotine-free e-liquid<br>• To get a higher nicotine concentration (over 20mg/mL)<br>• Less expensive than regular e-liquid<br>• Convenience of a bigger bottle<br>• To get a custom flavour or PG/VG mix<br>• Other<br>• Don’t know<br>Refusals were excluded
Vaping status:
Vaping status was constructed from two questions: “Have you ever tried an e-cigarette/vaped, even one or two puffs?” and ”When was the last time you used an e-cigarette/vaped?”.<br>Responses were coded:<br>• Vaped in past 30-days<br>• Ever vaped (ever tried vaping but not vaped in the past 30 days),<br>• Never vaped<br>Refusals were excluded
Smoking status
Smoking status was constructed from three questions: “Have you ever tried cigarette smoking, even one or two puffs?”, “How many cigarettes have you smoked in your entire life?” and “When was the last time you smoked a cigarette, even one or two puffs?”.<br>Responses were coded:<br>• Currently smoke (smoked in the past 30 days and smoked 100 or more cigarettes in lifetime)

• Ever smoked (tried smoking but smoked less than 100 cigarettes in lifetime or not smoked in the past 30 days)<br>• Never smoked<br>Refusals were excluded
Dual use
Smoking and vaping variables were combined from the responses detailed above<br><br>Responses were coded:<br>• Dual use (currently smoke, and vaped in the past 30 days)<br>• Exclusively vape (vaped in the past 30 days, and ever or never smoked)<br>• Exclusively smoke (currently smoke, and ever or never vaped)<br>• Formally smoked/vaped (ever smoked and vaped, or ever smoked and never vaped, or ever vaped and never smoked)<br>• Never smoked/vaped (never smoked and never vaped)
Nicotine concentration
Youth who had vaped in the past 30-days were asked, “Do the e-cigarettes, cartridges, pods, or e-liquids you currently use contain nicotine?”.<br>Those who responded ‘No’ were coded ‘0%(0 mg/mL)’.<br>If participants responded ‘Yes’, they were asked “How much nicotine do the e-cigarettes, cartridges, pods, or e-liquids you currently use contain?” with categories as percentages or mg/mL, in 1% (10mg/mL) increments.<br>If they responded ‘2-2.9%(20-29mg/mL)’, a clarification question asked, “You said 2% to 2.9% (20-29mg/mL). Would that be…” with options 2.0% (20mg/mL), or 2.1-2.9% (21-29mg/mL).<br><br>Responses were coded:<br>• 0% (0 mg/mL)<br>• 0.1-1.9% (1-19mg/mL)<br>• 2% (20mg/ml)<br>• 2.1% (21 mg/mL or more)<br>• Don’t Know<br>Refused’ were removed.

Supplementary table 2: Awareness and use of short-fills by respondent characteristics. ITC Youth Survey, England, 2021, weighted %(n)							
Outcome		A. Aware of short-fills			B. Used a short-fill in the past 30 days		
Sample	Total N=4224	Among all respondents (n=4224)			Among youth who vaped in the past 30-days (n=747)		
	%(n)	%(n)	AOR (95% CI)	p	%(n)	AOR (95% CI)	p
**Model 1**							
**Total**		23.0(974)			22.1(150)		
Vaping							
Never vaped	57.5(2429)	18.4(471)	1	Ref	-	-	-
Ever vaped	24.8(1048)	21.8(219)	0.93(0.73-1.18)	.550	-	-	-
Vaped in past 30-days	17.7(747)	41.9(284)	2.14(1.67-2.74)	**<.001**	22.1(150)	-	-
Smoking							
Never smoked	58.9(2486)	18.1(471)	1	Ref	5.3(6)	1	
Ever smoked	33.9(1433)	27.0(370)	1.30(1.05-1.61)	**.015**	19.5(83)	4.58(1.60-13.14)	**.005**
Currently smoke	7.2(305)	50.9(133)	3.13(2.26-4.33)	**<.001**	43.2(61)	13.7(4.56-40.93)	**<.001**
Age group							
16-17	43.7(1845)	18.4(391)	1	Ref	21.5(59)	1	
18-19	56.3(2379)	27.6(583)	1.52(1.27-1.80)	**<.001**	22.6(91)	1.02(0.63-1.65)	.942
Sex							
Male	34.5(1455)	23.6(514)	1	Ref	30.2(100)	1	
Female	65.5(2769)	22.3(460)	0.91(0.77-1.08)	.272	14.5(50)	0.40(0.26-0.62)	**<.001**
Race/ethnicity							
White	68.0(2873)	23.0(655)	1	Ref	23.6(120)	1	
All other racial/ethnic groups combined	32.0(1351)	23.0(289)	1.12(0.93-1.35)	.231	17.8(30)	0.99(0.58-1.72)	.990
**Model 2**	Among youth who had vaped in the past 30-days (*n=747*)				Among youth who had vaped in the past 30-days (*n=747*)		
Nicotine concentration							
0% (0mg/ml)	40.7(304)	31.8(88)	**1**	**Ref**	11.9(33)	1	Ref
0.1%-1.9%(1-19mg/ml)	27.6(206)	54.1(98)	1.68(1.06-2.69)	**.029**	32.5(59)	2.40(1.32-4.39)	**.004**
2.0% (20mg/ml)	8.0(60)	45.8(23)	1.41(0.74-2.69)	.294	31.7(15)	2.64(1.14-6.13)	**.024**
≥2.1% (≥21mg/ml)	11.4(85)	61.7(55)	2.22(1.23-3.99)	**.008**	40.8(37)	3.16(1.53-6.49)	**.002**
Don't know	12.3(92)	24.3(24)	0.59(0.31-1.10)	.097	7.4(6)	3.16(1.53-6.49)	.119
Vaping							
Never vaped	-	-	-	-	-	-	-
Ever vaped	-	-	-	-	-	-	-
Vaped in past 30-day	100(747)	41.9(284)	-	-	22.1(150)	-	-
Smoking							
Never smoked	15.1(113)	19.7(22)	1	Ref	5.3(6)	1	Ref
Ever smoked	63.5(474)	40.0(171)	2.63(1.40-4.95)	**.003**	19.5(83)	3.92(1.32-11.69)	**.014**
Currently smoke	21.4(160)	64.7(91)	5.97(2.88-12.35)	**<.001**	43.2(61)	9.16(2.90-29.00)	**<.001**
Age group							
16-17	32.7(244)	36.7(100)	1	Ref	21.5(59)	1	Ref
18-19	67.3(503)	45.4(183)	1.38(0.93-2.06)	.109	22.6(91)	0.94(0.57-1.56)	.814
Sex							
Male	31.6(236)	53.2(175)	1	Ref	30.2(100)	1	Ref
Female	68.4(511)	31.2(109)	0.40(0.28-0.59)	**<.001**	14.5(50)	0.43(0.27-0.68)	**<.001**
Race/ethnicity							
White	72.6(542)	42.6(217)	1	Ref	23.6(120)	1	Ref
All other racial/ethnic groups combined	27.4(205)	39.6(66)	1.15(0.76-1.74)	.515	17.8(30)	0.91(0.53-1.57)	.743
All analyses were weighted<br>Model 1 was adjusted for age, sex, race/ethnicity, smoking, vaping<br>Model 2 included only youth who had vaped in the past 30-days and was adjusted for age, sex, race/ethnicity, smoking and nicotine concentration.							

Supplementary table 3: Associations between awareness of short-fills and dual use status. ITC Youth Survey, England, 2021, weighted			
	Aware of short-fills N=4224		
	%(n)	AOR (95% CI)	p
**Dual status**			
Dual use	64.7(92)	1	Ref
Exclusively vape	35.9(192)	0.31(0.20-0.47)	**<.001**
Exclusively smoke	34.7(42)	0.29(0.17-0.50)	**<.001**
Formally smoked/vaped	20.4(265)	0.14(0.09-0.21)	**<.001**
Never smoked/vaped	17.9(383)	0.13(0.09-0.19)	**<.001**
Adjusted for age, sex and ethnicity<br>All analysis were weighted			

Supplementary table 4: Reasons for short-fill use among those who have used short-fills in the past 30 days, ITC Youth Survey, England, 2021, weighted %(n)					
	Use a nicotine free e-liquid	Higher nicotine concentration	Less expensive	Bigger bottle	Custom flavour or PG/VG
	%(n)	%(n)	%(n)	%(n)	%(n)
**Total**	25.4(38)	27.6(41)	37.6(56)	45.0(67)	34.5(52)
Age group					
16-17 years	26.0(15)	24.8(15)	53.0(31)	49.5(29)	40.8(24)
18-19 years	25.1(23)	28.3(27)	27.8(25)	42.0(38)	30.4(28)
Sex					
Male	20.7(20)	33.2(33)	38.1(38)	48.9(48)	41.4(41)
Female	34.8(18)	16.5(8)	36.7(18)	37.3(19)	20.8(11)
Race/ethnicity					
White	24.1(29)	22.8(27)	37.9(45)	50.1(60)	35.8(43)
All other racial/ethnic groups combined	30.6(9)	46.6(14)	36.4(11)	24.4(7)	29.2(9)
Smoking					
Never smoked	57.4(3)	26.3(20)	16.6(1)	0.0(0)	0.0(0)
Ever smoked	57.4(18)	18.0(1)	36.9(31)	47.8(40)	30.0(25)
Current smoked	27.2(17)	33.2(20)	40.6(25)	45.4(27)	43.8(27)
Usual nicotine concentration					
0% (0mg/ml)	42.3(14)	5.0(2)	43.1(14)	41.0(13)	20.8(7)
0.1%-1.9%(1-19mg/ml)	28.5(17)	27.6(16)	34.3(20)	55.7(33)	37.9(22)
2.0% (20mg/ml)	19.9(5)	34.4(17)	36.6(18)	32.7(16)	39.9(20)
≥2.1% (≥21mg/ml)	27.5(1)	70.6(2)	71.5(3)	42.1(2)	27.5(1)
Don't know	18.4(1)	69.4(4)	28.5(2)	62.0(3)	35.9(2)
All analyses were weighted<br>Participants could provide multiple responses					
